# Use of patient derived urine renal epithelial cells to confirm pathogenicity of *PKHD1* alleles

**DOI:** 10.1186/s12882-020-02094-z

**Published:** 2020-10-15

**Authors:** Elisa Molinari, Shalabh Srivastava, Rebecca M. Dewhurst, John A. Sayer

**Affiliations:** 1 Translational and Clinical Research Institute, Faculty of Medical Sciences, Newcastle University, International Centre for Life, Central Parkway, Newcastle upon Tyne, NE1 3BZ UK; 2 Renal Services, The Newcastle Hospitals NHS Foundation Trust, Newcastle upon Tyne, NE7 7DN UK; 3 NIHR Newcastle Biomedical Research Centre, Newcastle upon Tyne, UK

**Keywords:** Urine, ARPKD, Synonymous variant, Cilia

## Abstract

**Background:**

*PKHD1* is the main genetic cause of autosomal recessive polycystic kidney disease (ARPKD), a hereditary hepato-renal fibrocystic disorder which is the most important cause of end-stage renal disease during early childhood. ARPKD can also present in adulthood with milder phenotypes. In this study, we describe a 24-year-old woman with atypical polycystic kidney, no family history of renal disease and no obvious extra-renal manifestations who was referred for genetic investigation.

**Methods:**

We used a combination of next generation sequencing, Sanger sequencing and RNA and microscopy studies performed on urine-derived renal epithelial cells (URECs) to provide a genetic diagnosis of ARPKD.

**Results:**

A next generation sequencing panel of cystic ciliopathy genes allowed the identification of two heterozygous sequence changes in *PKHD1* (c.6900C > T; p.(Asn2300=) and c.7964A > C; p.(His2655Pro)). The pathogenicity of the synonymous *PKHD1* variant is not clear and requires RNA studies, which cannot be carried out efficiently on RNA extracted from proband blood, due to the low expression levels of *PKHD1* in lymphocytes.

Using URECs as a source of kidney-specific RNA, we show that *PKHD1* is alternatively spliced around exon 43, both in control and proband URECs. The variant p.(Asn2300=) shifts the expression ratio in favour of a shorter, out-of-frame transcript. To further study the phenotypic consequence of these variants, we investigated the ciliary phenotype of patient URECs, which were abnormally elongated and presented multiple blebs along the axoneme.

**Conclusions:**

We confirm the power of URECs as a tool for functional studies on candidate variants in inherited renal disease, especially when the expression of the gene of interest is restricted to the kidney and we describe, for the first time, ciliary abnormalities in ARPKD patient cells.

## Background

Autosomal recessive polycystic kidney disease (ARPKD; MIM # 263200) is a hereditary hepato-renal fibrocystic disorder, with an estimated incidence of 1:20,000 live births. Affected individuals typically present in utero with enlarged, echogenic kidneys and oligohydramnios. 30–50% of ARPKD patients die shortly after birth from respiratory insufficiency due to pulmonary hypoplasia. Children who survive the perinatal period have varied clinical presentations. Approximately one half develop end-stage renal failure. Cysts in the kidney mainly of collecting duct origin are often accompanied by biliary dysgenesis, hepatic fibrosis and portal hypertension, which can cause hypersplenism. There is no direct correlation between the renal and the hepatic phenotype. In rare cases, both renal and hepatic involvement may present in late adolescence and adult patients with a relatively mild clinical course have also been described. The diagnosis and treatment of adult ARPKD patients can be particularly challenging partly due to non-typical disease manifestations [1–3].

Mutations in the *PKHD1* gene are the primary cause of ARPKD [4–6]. *PKHD1* is a large gene that spans more than 469 kilobases of genomic sequence and encodes a single-transmembrane protein, called fibrocystin/polyductin (PKHD1), with a fairly restricted expression mainly limited to the kidney, liver, pancreas and testis [4, 5, 7, 8]. PKHD1 has been shown to localise to primary cilia and basal bodies [7, 9–12]. Some reports also describe an apical membrane, cytoplasmic and sub-apical localisation partially overlapping with the Golgi in tubular epithelial cells and the presence of PKHD1 in exosome-like vesicles [8, 11, 13]. It has been proposed a receptor function for PKHD1, on the basis of its structure and extracellular motif, but the ligand is unknown. Evidence that the large extracellular domain is shed in the extracellular space led also to speculations that PKHD1 may work as a bidirectional signalling molecule [4, 12]. PKHD1 was also shown to be involved in centrosome duplication and mitotic spindle assembly during cell division [14]. However, the exact functions of PKHD1 and the mechanism that links it to renal and hepatic fibrocystic disease are not clear.

The longest open reading frame of *PKHD1* comprises at least 66 exons but several lines of evidence indicate that *PKHD1/Pkhd1* undergoes a complex pattern of alternative splicing [4–6, 15–17]. The functional meaning of *PKHD1* splicing isoforms and whether these are translated into the corresponding proteins has not been definitely proven and results of immunoblotting to detect protein isoforms have been inconsistent across different studies [7, 10–13]. Moreover, the multiple PKHD1 protein isoforms reported by immunoblot maybe the result of post-translational processing of a single splicing isoform rather than that of the translation of multiple splicing products.

Mutations have been reported across the entire length of the gene [18]. Genotype-phenotype correlations have been proposed, based on the type of *PKHD1* mutations, with biallelic truncating mutations causing perinatal death and at least one hypomorphic missense mutation being necessary but not sufficient for survival [19]. However, exceptions to this rule have been described [17, 20].

It was proposed that *PKHD1* alternate splicing may contribute to the definition of ARPKD patients’ phenotype. Nonsense-mediated alternative splicing or basal exon skipping may lead to a circumvention of nonsense-mediated decay induced by truncating mutations, giving rise to unexpectedly mild phenotypes for patients carrying biallelic severe mutations. At the same time, missense or synonymous variants may affect the expression of specific isoforms, perhaps disrupting a critical stoichiometric and temporal balance between different protein products, giving rise to severe phenotypes [16, 17].

Functional investigations into the splicing isoforms of *PKHD1* is considerably hampered by the lack of well-validated immunoreagents as well as by the tissue-restricted expression pattern of *PKHD1*. The lack of *PKHD1* expression in tissues that are usually available for analysis such as lymphocytes or fibroblasts also poses a limit to functional studies on variants of unknown significance in this gene.

We recently described the use of urine-derived renal epithelial cells (URECs) as a patient-specific liquid biopsy of the kidney to investigate a variant that affects a kidney-specific alternate splicing [21]. Here we use URECs to carry out RNA studies to validate the pathogenicity of a synonymous variant in *PKHD1* and confirm a genetic diagnosis in an adult patient with atypical cystic kidney disease. We also provide a proof of principle of the utility of URECs to investigate ARPKD phenotypes at a cellular level, reporting for the first time ciliary defects in ARPKD patient cells.

## Methods

### Study approval

Ethical approval was given by the National Research Ethics Service Committee North East – Newcastle and North Tyneside 1 (08/H0906/28 + 5) and the National Research Ethics Service (NRES) Committee North East (14/NE/1076). All methods were performed in accordance with the relevant ethical guidelines and regulations.

Following informed and written consent, blood samples were obtained from affected patient, her relatives and unrelated wild type controls.

Following informed and written consent, human urine-derived renal epithelial cells (hURECs) were isolated from urine collected from ARPKD patient and two unrelated wild type controls and cultured as previously described [22, 23]. Briefly, cells were isolated from urine samples through repeated centrifugation passages and kept for the first 96 h in DMEM/high glucose and Ham’s F12 nutrient mix (1:1), supplemented with 10% (vol/vol) FBS, 140 U/ml penicillin, 140 μg/ml streptomycin, 3.5 μg/ml amphotericin B and REGM SingleQuot kit supplements (Lonza), at 37 °C in a humidified atmosphere of 5% (v/v) CO_2_. 96 h after isolation, medium was changed to Renal Epithelial Cell Growth Basal Medium (REBM, Lonza) supplemented with 0.5% FBS, 140 U/ml penicillin, 140 μg/ml streptomycin, 3.5 μg/ml amphotericin B and REGM SingleQuot kit supplements (Lonza). For ciliogenesis assays, cells were cultured in the same medium devoid of FBS for 48 h.

### Clinical and DNA sequencing

Clinical, imaging data and familial information was obtained by review of clinical records. DNA sequencing was performed using NHS cystic kidney disease NGS gene panel (East Anglian Medical Genetics Service). Confirmation of NGS findings and segregation studies were performed by Sanger sequencing.

### Immunofluorescence imaging

URECs were fixed in ice-cold methanol for 10 min. After 30 min saturation with 5% BSA in PBS, cells were incubated for 1 h at room temperature with the following primary antibodies in blocking solution: rabbit anti-ARL13B (Proteintech, 17711–1-AP); mouse anti-acetylated α-tubulin (Sigma T6793); mouse anti-ARL13B (Proteintech, 66739-1-Ig); rabbit anti-IFT88 (Proteintech, 13967-1-AP). Following washes in PBS, cells were incubated at room temperature for 1 h with the following secondary antibodies: donkey anti-rabbit Alexa Fluor 488 (Thermo Fisher); donkey anti-mouse Alexa Fluor 594 (Thermo Fisher). Following further washes in PBS, cells were incubated overnight at 4 °C with primary rabbit anti-Pericentrin antibody (Abcam ab 4448) directly labelled using Zenon Alexa Fluor 647 rabbit IgG labelling kit (Thermo Fisher), washed with 0.1% PBS Tween and post-fixed with 4% PFA for 15 min. Following final washes in PBS, cells were mounted in Vectashield (Vector Laboratories Ltd., H-1200). Images and *z*-stacks were captured in a blinded fashion, using a Nikon (A1) confocal inverted microscope. Each experiment was performed in duplicate.

### Image analysis

Following capture, images were analysed using FIJI (ImageJ) software. The length of cilia was measured using the segmented line tool on a maximum intensity projection of a *z*-stack, merge of ARL13B and acetylated α-tubulin channels was used to identify the cilia and ARL13B was used to quantify cilia length. Fluorescence intensity of ciliary IFT88 staining was measured as mean grey value on a sum of slices projection of a *z*-stack. To correct for local background intensity, the background fluorescence intensity was measured and subtracted.

### RNA preparation and RT-PCR

Total RNA from URECs and blood samples was isolated using RNeasy mini kit (Qiagen) according to the manufacturer’s instructions and quantified using a NanoDrop 2000 spectrophotometer. 250 ng or 1μg RNA were reverse-transcribed using an oligo-dT primer and SuperScript III Reverse Transcriptase (Thermo Fisher Scientific). The resulting cDNA was used pure in RT-PCR reactions and diluted 10-fold in nuclease-free water for qPCR experiments.

RT-PCR, using GoTaq® DNA Polymerase (Promega) and *PKHD1* gene and transcript-specific primer pairs, was performed to identify splice products of *PKHD1* in leukocytes and URECs. Amplification of *HPRT1* housekeeping gene cDNA was performed alongside.

Quantitative analysis of *PKHD1* transcripts was carried out using SYBR Green PCR Master Mix (Applied Biosystems) on a QuantStudio™ 7 Flex Real-Time PCR System (Applied Biosystems). Ct values for each sample are calculated as the arithmetic mean of three technical replicates. Expression levels were normalised to the expression of housekeeping genes *HPRT1* and *GUSB*.

Two biological replicates were used for control URECs.

### T-cloning and colony PCR

Competent *E. coli* cells were transformed with control and ARPKD URECs RT-PCR products ligated into pGEM-T Easy Vector (Promega) and plated onto LB/ampicillin/IPTG/X-Gal plates, to allow for white/blue screening of transformants for inserts. Colony PCR using GoTaq® DNA Polymerase (Promega) was performed on white colonies from both wild type control and ARPKD ligations and PCR products were Sanger sequenced.

### Primers

Gene and transcript-specific oligonucleotide primer sequences for PCR, RT-PCR and RT-qPCR were designed using Primer3 [24, 25] (Additional Table 1).

### Statistics

All data are shown as the mean ± standard deviation, unless otherwise stated. Unpaired t test or Fisher’s exact test was performed. A *P* value of less than 0.05 was considered statistically significant.

## Results

### Clinical features and genetic investigations of a patient with atypical polycystic kidney disease

**Fig. 1:**
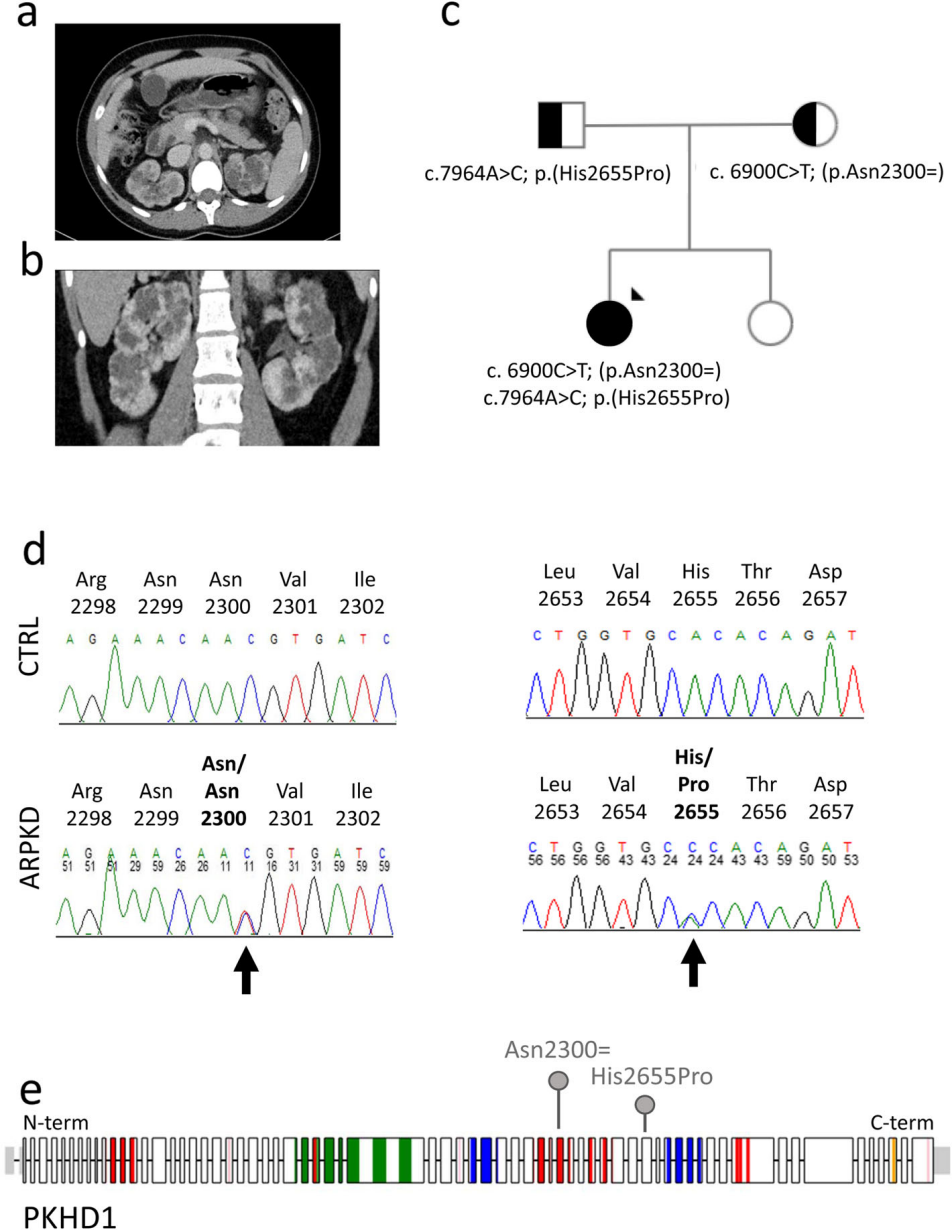
Clinical and genetic features of the proband. **a-b** Abdominal CT scan demonstrating bilateral renal cysts in (**a**) transverse and (**b**) coronal sections. No liver cysts are observable and spleen size is within normal limits. **c** Pedigree diagram showing segregation of the two variants found in the proband (arrow). **d** Sequence chromatograms showing compound heterozygous changes in *PKHD1* c.6900C > T; p.(Asn2300=) (predicted to affect transcript splicing) and c.7964A > C; p.(His2655Pro) in the proband (ARPKD), compared to wild type control (CTRL). **e** Schematic of the PKHD1 protein encoded by *PKHD1* gene (NM_138694, transcript ENST00000371117). Exon boundaries, protein domains and localisation of variants p.(Asn2300As=) and p.(His2655Pro) are displayed. Schematic was drawn using ProteinPaint [26] and SMART domain [27]. Protein domains, parallel beta-helix repeats, red; low complexity regions, pink; IPT domains, green; G8 domains, blue; transmembrane region, orange

The proband presented at the age of 24 years with tiredness and was found to have evidence of chronic kidney disease with an eGFR of 30 ml/min/1.73m^2^. Investigations including an abdominal ultrasound scan (USS) and CT scan revealed polycystic kidneys, with a normal appearance of liver and spleen (Fig. 1a, b). There was no family history of cystic kidney disease. She had a younger sister who was fit and well and both parents were well with normal abdominal USS. The pattern of cystic kidney disease was atypical and prompted further genetic investigations to look for autosomal recessive forms of polycystic kidney disease. Consistent with the clinical features, a next generation sequencing (NGS) panel of cystic ciliopathy genes allowed the identification of two heterozygous sequence changes in *PKHD1* (NM_138694; c.6900C > T; p.(Asn2300=) and c.7964A > C; p.(His2655Pro)). Segregation studies indicate that these variants are present in trans in the proband, with the variant p.(Asn2300=) inherited from the mother and the variant p.(His2655Pro) inherited from the father (Fig. 1c, d, e) (Table 1).

The variant p.(His2655Pro) is listed as a likely disease-causing variant on Human Gene Mutation Database (HGMD) [37] and has been reported previously in an unrelated individual with a diagnosis of ARPKD who also carried a nonsense variant on the opposite allele [35]. The nucleotide substitution alters a highly conserved amino acid and there is a moderate physio-chemical difference between the wild type and the variant residue. Another nucleotide substitution at the same position, which results in a different missense change, c.7964A > G; p.(His2655Arg), is also listed as pathogenic on HGMD in association with ARPKD, further underlining the functional importance of this region of the protein [36]. The majority of in silico analysis algorithms also predict that the c.7964A > C variant is disease-causing (Table 1).

**Table 1:** *PKHD1* variants details

Variant	Molecular consequence	db SNP ID	Accession number (ClinVar or HGMD)	Clinical significance on ClinVar/HGMD	Allele frequency (GnomADe)	SIFT [28]	PolyPhen [29]	CADD [30]	REVEL [31]	References
**c.6900C > T; (p.Asn2300=)**	synonymous	rs776060304	VCV000558073	Uncertain significance	T = 0.000016 (4/251312)	N/A	N/A	9 (likely benign)	N/A	[32–34]
**c.7964A > C; p.(His2655Pro)**	missense	rs748196998	CM163038	Likely disease-causing	C = 0.000004 (1/251258)	0 (deleterious)	0.994 (probably damaging)	24 (likely benign)	0.819 (likely disease causing)	[35]
c.6900C > G; (p.Asn2300Lys)	missense	rs776060304	VCV000552623	Uncertain significance	N/A	0 (deleterious)	0.999 (probably damaging)	22 (likely benign)	0.489 (likely benign)	[36]
c.7964A > G; p.(His2655Arg)	missense	N/A	CM1612097	Pathogenic	N/A	0 (deleterious)	0.990 (probably damaging)	24 (likely benign)	0.786 (likely disease causing)	[36]

Molecular consequence, db SNP ID, variant accession number, clinical significance, allele frequency and pathogenicity prediction values according to a range of different algorithms for the two variants identified in the proband (c.6900C > T (p.Asn2300=); 7964A > C; p.(His2655Pro), in bold) and two different variants described in the literature that change the same nucleotides (c.6900C > G (p.Asn2300Lys); c.7964A > G; p.(His2655Arg))

The clinical significance of the second *PKHD1* variant p.(Asn2300=) is less certain. It has a low population frequency in the gnomAD cohort [38] (AFR: 0.006%), but the altered nucleotide is weakly conserved and the substitution does not alter the protein sequence. The variant had been previously reported to be disease causing in a compound heterozygous individual with a missense variant on the other allele, prenatally presenting with multicystic kidneys [32] (Table 1).

### URECs as a tool to perform RNA studies on *PKHD1*

**Fig. 2:**
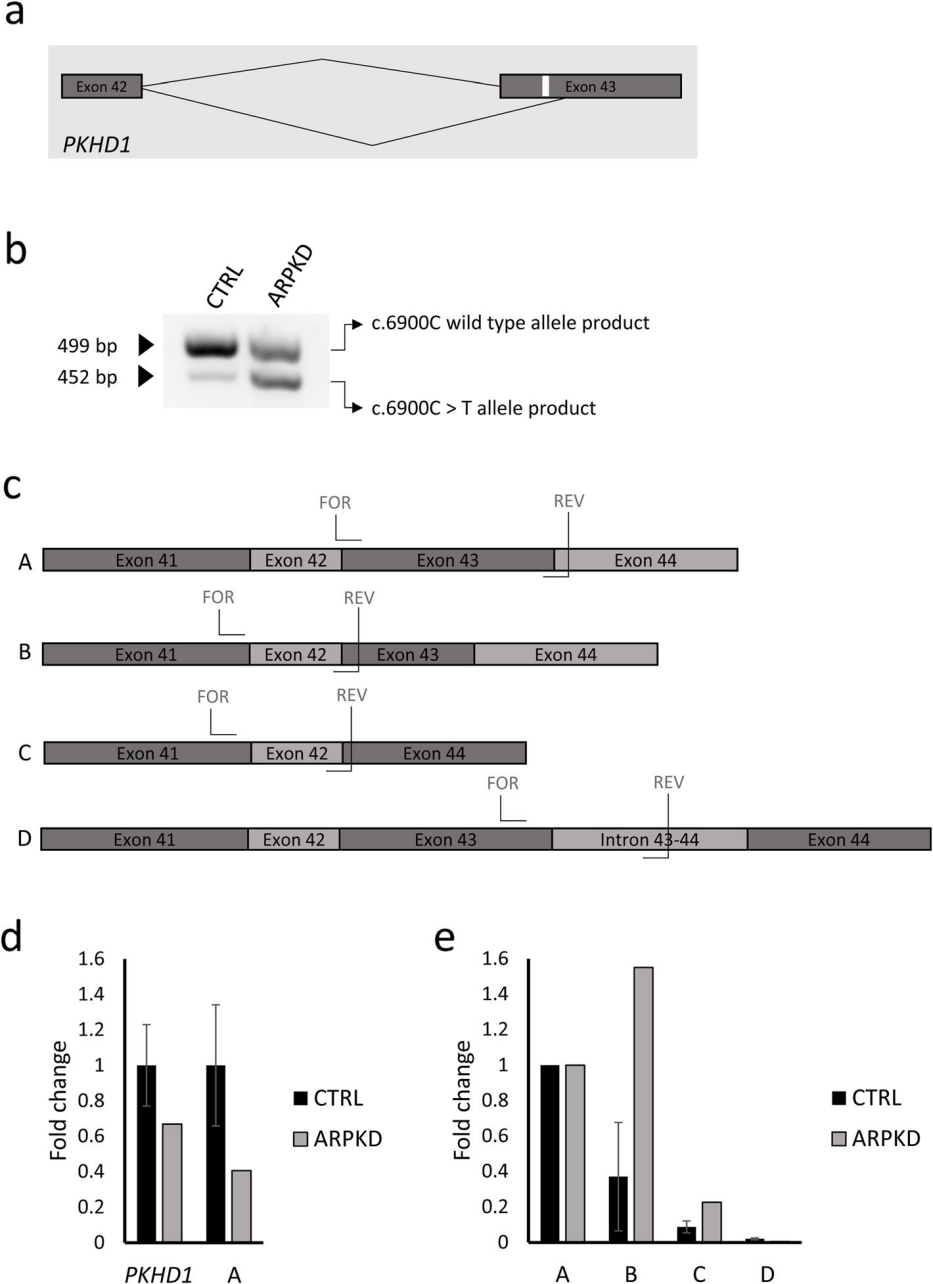
ARPKD synonymous variant c.6900C > T promotes alternate splicing of exon 43 above basal level. **a** Schematic of human *PKHD1* exon 42 and exon 43, that illustrates the alternate splicing of exon 43, promoted by the variant c.6900C > T (in white), leading to a splicing product devoid of the first 47 nucleotides of exon 43. **b** RT-PCR on cDNA isolated from wild type control URECs and ARPKD URECs lysates demonstrates alternate splicing of exon 43 in both cell lines. **c** Schematic of alternate splicing products of human *PKHD1* around exon 43 predicted to occur on the basis of Sanger sequencing of colony PCR products after T cloning of URECs RT-PCR products. Position of each transcript-specific primer set is specified within this schematic. Transcript A contains a full-length exon 43 (131 nt), transcript B contains a shorter exon 43, devoid of the first 47 nucleotides, transcript C is completely devoid of exon 43 and transcript D contains a full-length exon 43 and a portion (124 nt) of intron 43–44. **d** *PKHD1* gene expression analysis of control and ARPKD URECs. Expression level of *PKHD1*, as measured by using a set of primers that do not differentiate between different splicing products around exon 43, are reduced by 30% in ARPKD URECs compared to the wild type control. Levels of transcript A, containing full-length exon 43, are reduced by 60% in ARPKD URECs compared to the wild type control. The bars represent the mean values from the expression levels in two different wild type control UREC lines and the expression levels in ARPKD URECs, respectively. Values are normalised to wild type controls mean values. **e** Expression analysis of the different transcript isoforms (transcript B, C and D) around exon 43 of *PKHD1*, normalized to the expression levels of transcript A, in control and ARPKD URECs. Transcript B is upregulated 1.5-fold in ARPKD URECs compared to wild type. Expression levels of transcript B and C are low (20% or less of the expression levels of transcript A) in both control and ARPKD URECs. The bars represent the mean values from the expression levels in two different wild type control UREC lines and the expression levels in ARPKD URECs, respectively

Previous studies indicated that the synonymous variant p.(Asn2300=) affects an exonic splicing enhancer and that the transition c.6900C > T causes aberrant mRNA splicing, leading to a shorter *PKHD1* transcript, devoid of the first 47 nucleotides (nt) of exon 43 and resulting in a reading frameshift [32] (Fig. 2a). However, this change is still listed as a variant of unknown significance on ClinVar (Variation ID 558073), as a functional ratification has not been convincingly shown.

To confirm the pathogenicity of the synonyous variant p.(Asn2300=) detected in the proband and verify that it affects the splicing of *PKHD1* in patient’s cells, we extracted RNA from whole blood of proband and of an unrelated wild type control. RT-PCR using primers designed to target a region around exon 43 of *PKHD1* failed to amplify the target cDNA sequence (Additional Figure 1).

We recently showed that urine-derived renal epithelial cells (URECs) can be used to study genetic variants that affect kidney-specific splicing [21]. Following a similar principle, we reasoned that we could use URECs as a source of RNA to perform studies on genes whose expression is mainly restricted to the kidney. Following a previously described protocol, we isolated and cultured non-transformed human urine-derived renal epithelial cells (URECs) from the proband. In contrast to whole blood cDNA, when we used an equivalent amount of cDNA from ARPKD URECs, we were able to obtain amplification of the target region of *PKHD1* by RT-PCR, indicating that the low expression levels of *PKHD1* in the blood (Table 2) may render the amplification by RT-PCR of the corresponding cDNA inefficient (Additional Figure 1).

**Table 2:** *PKHD1* gene expression data in different human tissues

Tissue	GTEx (TPM)	HPA (TPM)
Kidney	10.872	17.5
Pancreas	5.098	2.4
Testis	2.223	1.7
Liver	0.964	0.7
Skin	0.133	0.6
Blood	0.000	0.0

*PKHD1* (ENSG00000170927) gene expression data in the human tissues that show highest expression levels according to GTEx (kidney, pancreas, testis and liver,) and in skin and blood. The data (expressed as Transcript Per Million, TPM) were obtained from: the GTEx Portal [39] and dbGaP accession number phs000424.v8.p2 and the Human Protein Atlas [40] on 04/30/20

### Investigation of the effects of the synonymous variant p.(Asn2300=) on the splicing of *PKHD1* transcript in ARPKD URECs

RT-PCR on ARPKD URECs cDNA indeed revealed the presence of two splicing products. The molecular weight of the corresponding bands on gel electrophoresis, 499 bp and 452 bp, respectively, is consistent with the presence of a transcript containing a partially truncated exon 43 (lacking 47 nt), possibly resulting from the allele carrying the variant p.(Asn2300=) and a transcript with a full-length exon 43 (131 nt), possibly resulting from the other allele (Fig. 2b and Additional Figure 1).

In order to compare the splicing pattern of *PKHD1* in ARPKD cells to that of control cells, we isolated and cultured URECs from an unrelated wild type control.

Intriguingly, when we performed RT-PCR on RNA extracted from control URECs, we observed the presence of the same PCR products, running at 499 bp and 452 bp on gel electrophoresis (Fig. 2b), indicating that the skipping of 47 nucleotides of exon 43 occurs at a basal level in control URECs.

In order to verify that the two products detected on agarose gel corresponded to the expected splicing isoforms (one containing a full-length exon 43 and one devoid of the first 47 nt of exon 43), we performed a T-cloning of the control and ARPKD RT-PCR products, followed by PCR of the transformant *E. coli* colonies, to allow for the amplification of each RT-PCR product at a time (Additional Figure 2). The PCR products from different colonies separated into 4 different molecular weight bands on gel electrophoresis (623 bp, 499 bp, 452 bp and 368 bp) (Additional Figure 3a), indicating the presence of 4 different splicing isoforms of *PKHD1* around exon 43, in both control and ARPKD URECs. The detection of additional bands from colony PCR is probably due to the amplification of each cloned RT-PCR product at a time, while in the case of a RT-PCR directly performed on cDNA preparation, multiple *PKHD1* templates compete for the same reagents in the PCR reaction, possibly leading to an inefficient amplification of the least abundant ones. We then Sanger sequenced the colony PCR products. Analysis of the sequence chromatograms retrieved the presence of 4 different *PKHD1* splicing products around exon 43; a transcript A, which corresponds to the 499 bp band and contains a full-length exon 43, a transcript B, corresponding to 452 bp band and devoid of the first 47 nucleotides of exon 43 and two additional transcripts C and D, possibly products of minor alternate splicing, which correspond to 368 bp and 623 bp bands, respectively. Transcript C completely lacks exon 43 and transcript D contains a full-length exon 43 and a portion (124 nt) of intron 43–44 (Fig. 2c and Additional Figure 3b). Transcript B, C and D are predicted to contain a frameshift and to encode for a truncated PKHD1 protein.

Since the relative intensity of the two RT-PCR bands (499 bp and 452 bp), corresponding to *PKHD1* transcripts A and B, is different between control and ARPKD cells (Fig. 2b), we hypothesised that alternate splicing of exon 43 occurs at a basal level, but is promoted by the synonymous change p.(Asn2300=). To confirm this hypothesis, we designed 4 different sets of primers, specific for each splicing product predicted to occur on the basis of the Sanger sequencing of colony PCR products (transcripts A, B, C and D), tested them on the 4 different colony PCR products to verify their specificity (Additional Figure 4) and performed RT-qPCR on the RNA extracted from ARPKD URECs and from URECs derived from two different unrelated wild type controls.

RT-qPCR confirmed that ARPKD URECs express lower levels of total *PKHD1* (70%, as assessed using primers that do not differentiate among different splicing isoforms around exon 43) and lower levels (40%) of transcript A, containing a full-length exon 43, compared to the average abundance in the two unrelated controls (Fig. 2d, Additional Figure 5a).

RT-qPCR on control and ARPKD URECs, using transcript-specific primers for the 4 different splicing isoforms, revealed that the ratio between the truncated transcript B and the full-length transcript A is higher (1.5 versus 0.4-fold) in ARPKD URECs compared to controls. The expression levels of the additional splicing products C and D are low in both ARPKD and control URECs (Fig. 2e, Additional Figure 5b).

### Investigation of the ciliary phenotype in ARPKD cells

**Fig. 3:**
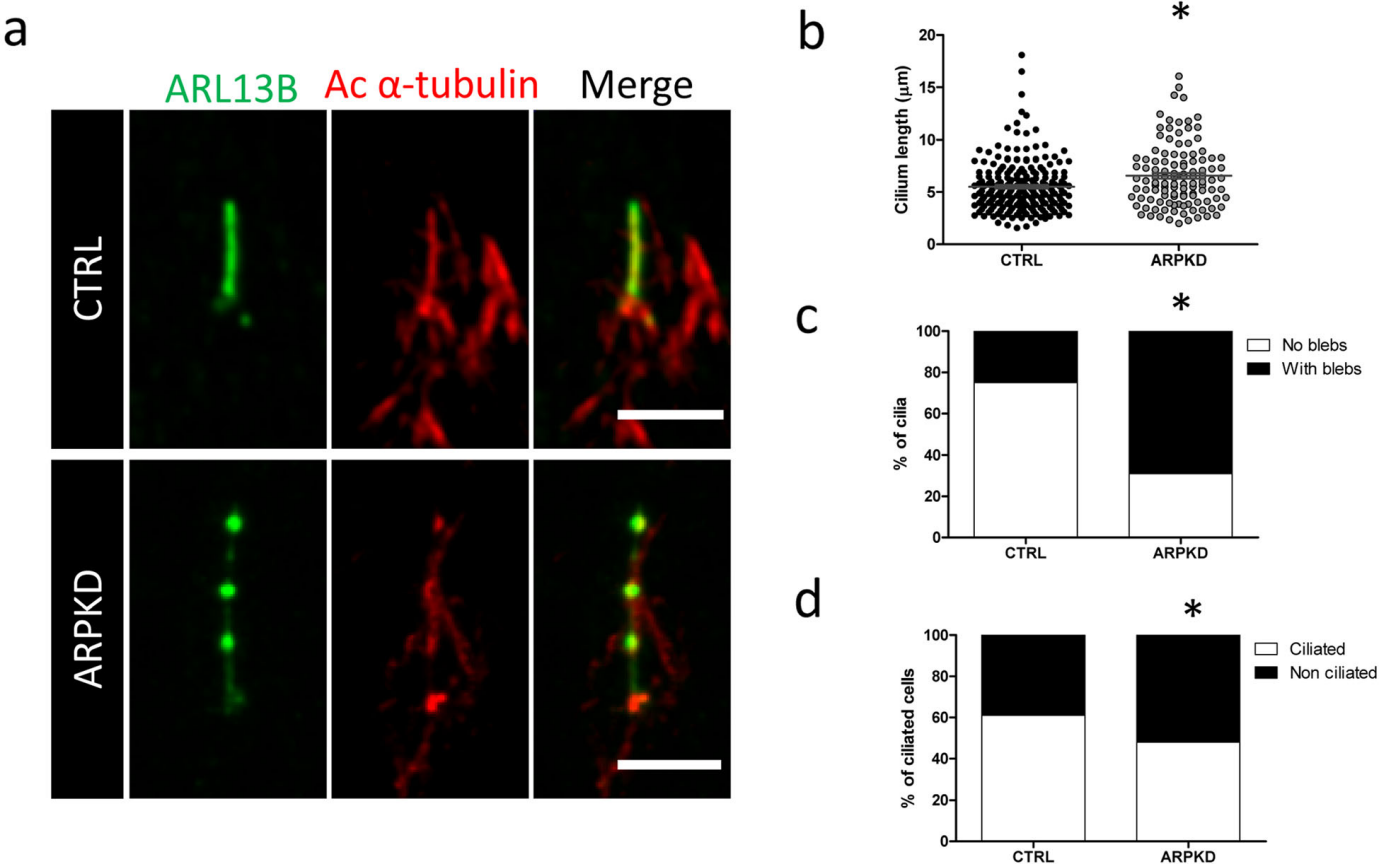
ARPKD URECs display an elongated ciliary phenotype. **a** Representative immunofluorescence images of wild type control URECs and ARPKD URECs, serum starved for 48 h. ARPKD cells display elongated cilia when compared to control, with swellings along the axoneme and at the ciliary tip. Green – ARL13B, Red – acetylated α-tubulin. Scale bar 5 μm. **b** Quantification of cilia length in control URECs and ARPKD URECs. The mean cilia length measured for control URECs was 5.5 μm. Mean length of ARPKD URECs was 6.5 μm. *P < 0.001, unpaired t test, *n* = 338. **c** Quantification of the percentage of cilia that display a bulbous phenotype with one or more blebs along the axoneme in control and ARPKD URECs. The percentage of cilia with blebs was 25% in control cells and 69% in ARPKD URECs. *P < 0.001, Fisher’s exact test, *n* = 229. **d** Quantification of percentage of ciliated cells in control URECs and ARPKD URECs. The percentage of ciliated cells for control cells was 61%. The percentage of ciliated cells measured for ARPKD cells was 48%. *P < 0.001, Fisher’s exact test, *n* = 694

Since PKHD1 was shown to localize to the primary cilium, a microtubule-based, hair-like structure that protrudes from the cell membrane and is thought to work as a critical sensory antenna [41], we set forth to investigate the ciliary phenotype of ARPKD URECs, as an additional investigation of the phenotype associated with these *PKHD1* alleles. Immunofluorescence analysis of cilia structure revealed elongated primary cilia on ARPKD URECs compared to control cells (Fig. 3a, b). We observed a bulbous ciliary phenotype (Fig. 3a, c), with several blebs along the axonemal membrane and a discontinuous staining for the ciliary membrane marker ARL13B and for the axonemal marker acetylated α-tubulin. Frequent swelling at the ciliary tip was also observed, as confirmed by ARL13B and pericentrin co-staining to define the proximo-distal ciliary axis (Additional Figure 6).

Swelling of ciliary axoneme had been previously associated to defects in the ciliary intraflagellar transport (IFT) [42, 43]. In order to study the integrity of IFT in ARPKD URECs, we stained the cells for the anterograde IFT complex component IFT88. Preliminary analysis of the localisation of IFT88 within ARPKD cilia did not show an obvious defect when compared to control cilia (Additional Figure 7a, b).

Quantification of ciliated cells revealed a reduced percentage of ARPKD cells that display a primary cilium after serum starvation compared to control URECs (Fig. 3d). Overall, these data confirm a ciliary phenotype in ARPKD URECs.

## Discussion

Here we describe a patient who presented with chronic kidney disease associated with atypical polycystic kidney and no family history of renal disease. There is an increasing awareness of the increasing number of genetic causes of atypical cystic kidney disease that may be both autosomal dominantly or recessively inherited. Adult presentations of nephronophthisis, ARPKD, renal cysts and diabetes syndrome, and atypical forms of autosomal dominant polycystic kidney disease (ADPKD) such as *GANAB* and *DNAJB11* [44, 45] now being diagnosed through increased availability of genetic testing through NGS panels, whole exome (WES) and whole genome sequencing (WGS).

The mild atypical cystic kidneys disease presentation in this case is consistent with mutations in *PKHD1*, however it is interesting that no signs of congenital hepatic fibrosis or other liver involvement are apparent, with normal platelet count also being observed in this patient. Patients with ARPKD may have cystic kidney disease without liver involvement, but in adult cases there is typically an increasing involvement of the liver with raised liver enzymes being seen in the majority of cases. Diagnostic NGS testing for cystic ciliopathy genes retrieved a likely pathogenic missense variant (c.7964A > C; p.(His2655Pro)) in *PKHD1*. A second synonymous variant (c.6900C > T; p.(Asn2300=)) in trans was also identified, which had been previously reported as disease-causing in an ARPKD patient [32].

In order to confirm a genetic diagnosis of ARPKD, we set forth to investigate the splicing effects of the synonymous variant c.6900C > T in *PKHD1* using URECs as a patient-specific liquid biopsy of the kidney for RNA studies.

Our results indicate that, in ARPKD URECs, exon 43 is alternatively spliced, producing transcripts predicted to contain premature termination codons. Unexpectedly, these transcripts were found to be expressed at a lower level also in control cells. Analysis of the sequence chromatograms from colony PCR retrieved the presence of 4 different *PKHD1* splicing products around exon 43. Transcript A contains a full-length exon 43, transcript B is devoid of the first 47 nucleotides of exon 43, transcript C is completely devoid of exon 43 and transcript D contains a full-length exon 43 and a short portion of intron 43–44. Transcript B, C and D are predicted to contain a frameshift and a premature stop codon. While transcript B and C are expressed at near-undetectable level, the abundance of transcript B is 40 and 150% of the expression observed for full-length transcript A in controls and ARPKD URECs, respectively. We conclude that alternate splicing of exon 43, producing transcript B, occurs at a basal level, but is promoted by the synonymous change p.(Asn2300=). The functional role of this alternate transcript is unknown. Since transcript B contains a premature stop codon and it is predicted to encode for a truncated protein, it may be subject to nonsense-mediated decay. The unproductive splicing of human and murine *PKHD1/Pkhd1* gene, leading to premature stop codon-containing transcripts, has been described in non-pathological conditions by several other reports and was shown to involve different exons [5, 15–17]. We hypothesise that this extensive unproductive splicing may participate to the regulation of the expression levels of *PKHD1*, through a mechanism of gene regulation known as alternative splicing-coupled nonsense-mediated decay (AS-NMD) [46–48]. Disturbance to this process, induced by splicing variants such as c.6900C > T p.(Asn2300=), can contribute to the pathogenicity of ARPKD, as previously shown for cancer and autoimmune diseases [49–51]. The synonymous variant p.(Asn2300=), by promoting the production of the premature stop codon-containing transcript B, may decrease the levels of *PKHD1* expression below a certain threshold required for its normal function. Indeed, we show that in ARPKD URECs the abundance of *PKHD1* transcript A is decreased to 40% of the expression levels in control URECs. Analysis of the effects of the inhibition of nonsense-mediated decay on the expression of transcript isoforms will help to better define their functional role.

The likely pathogenic missense variant p.(His2655Pro) on the second allele changes a highly conserved amino acid, which may be essential for the correct function of PKHD1. However, we cannot exclude a potential effect of this missense variant on *PKHD1* splicing.

This study underlines the importance of assessing the potential splicing effects of synonymous changes in known disease causing genes. The significance of synonymous variants in *PKD1* and *NPHP3*, underlying ADPKD and nephronophthisis, respectively, has recently been described [21, 52]. Interpretation of variants of unknown significance in WES and WGS datasets is a major challenge in the NGS era and often requires functional studies to assess their significance. At the same time, functional studies cannot always be carried out directly on patients’ primary material, when the gene of interest in not expressed in the peripheral tissues usually available for analysis such as lymphocytes or fibroblasts.

*PKHD1* is expressed at low levels in blood and skin fibroblasts (Table 2). Indeed, we show that amplification of *PKHD1* cDNA is not efficient using total RNA extracted from the blood.

We have previously shown that URECs can be a valuable source of RNA when studying genetic variants that affect kidney-specific splicing in cystic kidney disease-causing genes [21].

Using URECs as a tissue-relevant, patient-specific disease model we could obtain information on the aberrant splicing of *PKHD1*, whose expression is mainly restricted to the kidney, directly in patient cells, circumventing the necessity for more time-consuming and artefact-prone methods such as minigene constructs for splicing studies.

Furthermore, we employed URECs to study the functional effects on ciliary phenotype of *PKHD1* variants, since the protein product of *PKHD1* was shown to localise to cilia and basal bodies [7, 9–12], as the vast majority of genes associated to cystic kidney disease [53].

Despite its importance in human disease, little is known about the function of PKHD1 and this is the first time that ciliary defects are described in human ARPKD cells. Disruption of *PKHD1* was shown to cause cilia defects in orthologous rat and mouse models [54–56]. In PCK rats, which carry a splicing change in *Pkhd1* that causes skipping of exon 36 and a frameshift, cholangiocytes display structurally abnormal cilia with bulbous extensions of the ciliary tip or in the ciliary axonemal membrane and cilia length defects [54, 55]. Similarly, primary cilia in the affected biliary tree and proximal tubules of *Pkhd1*^*del2/del2*^ mice are misshapen, with multiple blebs [56]. These ciliary structural defects are very reminiscent of the bulbous ciliary phenotype that we describe in ARPKD URECs. We also observe an elongated ciliary phenotype and an impaired cilia biogenesis, previously described in other recessive cystic kidney disorders [57–61]. The striking lack of literature data on the possible functional role of *PKHD1* gene product makes it hard to identify a possible mechanism that explains the observed ciliary structural defects. The bulbuous phenotype may indicate an IFT dysfunction, but we could not observe an overt defect in the ciliary localisation of the anterograde IFT complex subunit IFT88. The implication of focal adhesion dysregulation in ARPKD-related PKHD1 deficiency [62] may suggest a possible involvement of PKHD1 in actin cytoskeleton dynamics, which is a known regulator of ciliogenesis [63]. Indeed, in mouse inner medullary collecting duct cells (IMCD), knock down of *PKHD1* was shown to cause ciliary defects as well as to perturb actin cytoskeleton organization and to increase the levels of active RhoA [64], which in turn regulates the polymerisation of actin filament [65]. Increased RhoA-GTP levels and actin disorganisation have been linked to ciliogenesis defects in other renal ciliopathy models [66]. More experiments will be required to verify whether PKHD1 depletion could induce ciliary defects through dysregulation of the actin cytoskeleton and whether these defects are a common signature in ARPKD. Only few previous studies investigated the ciliary phenotype of human ARPKD and did not report structural defects [67, 68]. Freedman et al. showed normal ciliary biogenesis and length in ARPKD induced-pluripotent stem cells, possibly highlighting a potential tissue-specificity of the ciliary phenotypes in ARPKD or different, variant-dependent pathogenic mechanisms [68].

Another possible explanation for this discrepancy is that the ciliary defect that we observe in ARPKD URECs is not a direct consequence of PKHD1 depletion, rather an effect of the complex tissue dysregulation that intervenes in the cystic kidney epithelium. Knock down of *PKHD1* in URECs derived from healthy donors will help clarify this important point and rescue experiments with wild type or mutant *PKHD1* alleles can provide a definitive confirmation of the direct link between the aberrant transcripts and the ciliary phenotype.

Interspecies differences between murine models and human, the low expression levels of *PKHD1* in the majority of tissues, coupled with possible tissue-specific effects of mutations and the lack of good immunoreagents, have so far led to inconsistent results and impaired a detailed investigation of the functions of PKHD1. Functional investigations and mechanistic studies on URECs derived from controls and ARPKD patients as an ex vivo disease-relevant model promise to shed new light on PKHD1 functions.

## Conclusions

With this study, we confirmed the utility of using URECs as a liquid biopsy of the kidney to carry out RNA and functional studies on kidney-specific genes or genes that are expressed at low levels in peripheral tissues. Using URECs directly derived from an adult patient with atypical polycystic kidney and no liver involvement, we validated the pathogenicity of a synonymous variant in *PKHD1* and confirmed a genetic diagnosis of ARPKD. Our RNA studies also contribute to shed light on the physiological alternate splicing of *PKHD1* in normal URECs, corroborating the evidence for a complex alternate splicing pattern of this gene. We also provide the first description of the ciliary phenotype in ARPKD URECs.

The availability of easy to obtain patient- and tissue-specific models for functional studies on variants of unknown significance is particularly critical in the NGS era. URECs represent an extremely powerful non-invasive personalised tool for such studies in inherited renal disorders.

Further functional studies on URECs derived from other ARPKD patients could also highlight common cellular and ciliary defects and help reveal the elusive function of PKHD1 in human kidney cells.

## Supplementary information

**Additional file 1 **: **Additional Table 1**. Oligonucleotide primers. **Additional Figure 1**. RT-PCR on URECs and blood cDNA. **Additional Figure 2**. Schematic of T-cloning of different RT-PCR products to allow for the amplification of each product at a time. **Additional Figure 3**. Colony PCR after T-cloning of RT-PCR products indicates the expression of 4 different *PKHD1* transcript isoforms around exon 43 in ARPKD URECs. **Additional Figure 4**. PCR using transcript-specific primer sets shows the specificity of each primer pair to amplify only the corresponding colony PCR product. **Additional Figure 5**. Arithmetic mean of ΔΔCt values of 3 technical replicates for each sample from *PKHD1* expression analysis shown in Fig. 2d and e. **Additional Figure 6**. Representative micrograph of ARPKD URECs. **Additional Figure 7**. IFT88 staining of CTRL and ARPKD URECs. **Additional Figure 8**. Full gel image of RT-PCR on cDNA isolated from wild type control URECs and ARPKD URECs lysates (Fig. 2b), demonstrating alternate splicing of exon 43 in both cell lines. **Additional Figure 9**. Full gel image of RT-PCR on URECs and blood cDNA (Additional Figure 1). **Additional Figure 10**. Full gel image of colony PCR after T-cloning of RT-PCR products (Additional Figure 3), showing the expression of 4 different *PKHD1* transcript isoforms around exon 43 in ARPKD. **Additional Figure 11**. Full gel image of PCR with transcript-specific primer sets (Additional Figure  4).

## Data Availability

The data generated and further information on the materials used in the current study are available from the corresponding author on request.

## Abbreviations

ADPKD: Autosomal dominant polycystic kidney disease
ARPKD: Autosomal recessive polycystic kidney disease
NGS: Next generations sequencing
URECs: Urine-derived renal epithelial cells
WES: Whole exome sequencing
WGS: Whole genome sequencing
