# Supplementary material for: Use of patient derived urine renal epithelial cells to confirm pathogenicity of PKHD1 alleles
Source: BMC Nephrol. 2020 Oct 15;21:435. doi: 10.1186/s12882-020-02094-z (PMC7559414; doi:10.1186/s12882-020-02094-z)
Supplement: Supplementary file 1 — Additional file 1 : Additional Table 1. Oligonucleotide primers. Additional Figure 1. RT-PCR on URECs and blood cDNA. Additional Figure 2. Schematic of T-cloning of different RT-PCR products to allow for the amplification of each product at a time. Additional Figure 3. Colony PCR after T-cloning of RT-PCR products indicates the expression of 4 different PKHD1 transcript isoforms around exon 43 in ARPKD URECs. Additional Figure 4. PCR using transcript-specific primer sets shows the specificity of each primer pair to amplify only the corresponding colony PCR product. Additional Figure 5. Arithmetic mean of ΔΔCt values of 3 technical replicates for each sample from PKHD1 expression analysis shown in Fig. 2d and e. Additional Figure 6. Representative micrograph of ARPKD URECs. Additional Figure 7. IFT88 staining of CTRL and ARPKD URECs. Additional Figure 8. Full gel image of RT-PCR on cDNA isolated from wild type control URECs and ARPKD URECs lysates (Fig. 2b), demonstrating alternate splicing of exon 43 in both cell lines. Additional Figure 9. Full gel image of RT-PCR on URECs and blood cDNA (Additional Figure 1). Additional Figure 10. Full gel image of colony PCR after T-cloning of RT-PCR products (Additional Figure 3), showing the expression of 4 different PKHD1 transcript isoforms around exon 43 in ARPKD. Additional Figure 11. Full gel image of PCR with transcript-specific primer sets (Additional Figure 4). [file 12882_2020_2094_MOESM1_ESM.pdf]

Additional Information

Use of patient derived urine renal epithelial cells to confirm pathogenicity of *PKHD1* alleles

Elisa Molinari, Shalabh Srivastava, Rebecca M. Dewhurst and John A. Sayer

Additional Table 1: Oligonucleotide primers

PKHD1 FOR	5’-GGTGGGAGCTATGAGAGAGTC-3’
PKHD1 REV	5’-GCCAGTGACATTATCCCAAGG-3’
PKHD1 ALL FOR/Transcript C FOR	5’-ATTTTAGGTCATGCGCTGCT-3’
PKHD1 ALL REV	5’-TCTTTCCTTCCATGAATTGCCT-3’
PKHD1 Transcript A FOR	5’-GACTGGTCAGGACATGGAAA-3’
PKHD1 Transcript B FOR	5’-TAGGTCATGCGCTGCTAGTT-3’
PKHD1 Transcript B REV	5’-CTCGGCACCAGAAACCATC-3’
PKHD1 Transcript C REV	5’-ACTCTGTTCCCCATCTTTCCT-3’
PKHD1 Transcript D FOR	5’-ATCTGCAGTCCCACCAATGT-3’
PKHD1 Transcript D REV	5’-GCCTTCTGCCGTGAGTAAAG-3’
HPRT1 FOR	5’-TGACACTGGCAAAACAATGCA-3’
HPRT1 REV	5’-GGTCCTTTTCACCAGCAAGCT-3’
GUSB FOR	5’-CCTGTGACCTTTGTGAGCAA-3’
GUSB REV	5’-AACAGATCACATCCACATACGG-3’

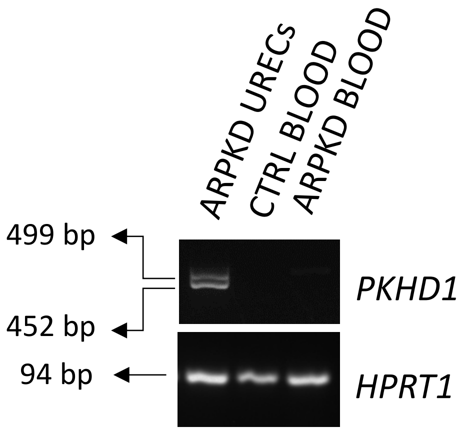

Additional Figure 1. RT-PCR on URECs and blood cDNA. *PKHD1* is expressed at low levels in lymphocytes (blood) and fails to be amplified by RT-PCR on ARPKD blood cDNA, but it is expressed at a detectable level in URECs.

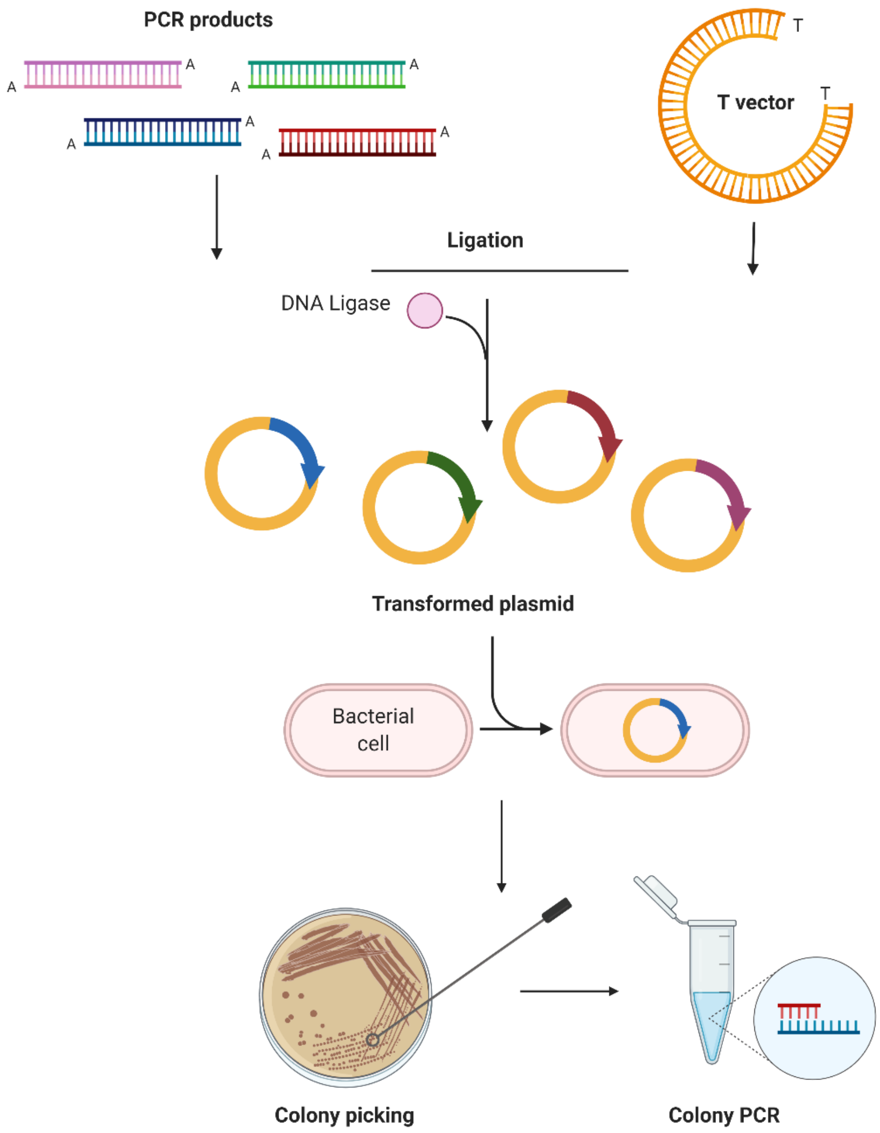

Additional Figure 2. Schematic of T-cloning of different RT-PCR products to allow for the amplification of each product at a time. Thermostable polymerases can add a single deoxyadenosine A to the 3´-ends of the amplified fragments. A vector that contains 3´-T overhangs at the insertion site provides compatible overhang for PCR products. Ligation generates transformed plasmids that contain one of the different RT-PCR products as an insert. Following transformation of competent cells and seeding on agarose plates, a single colony lysate can be used as a template for PCR. This image was created with BioRender.com.

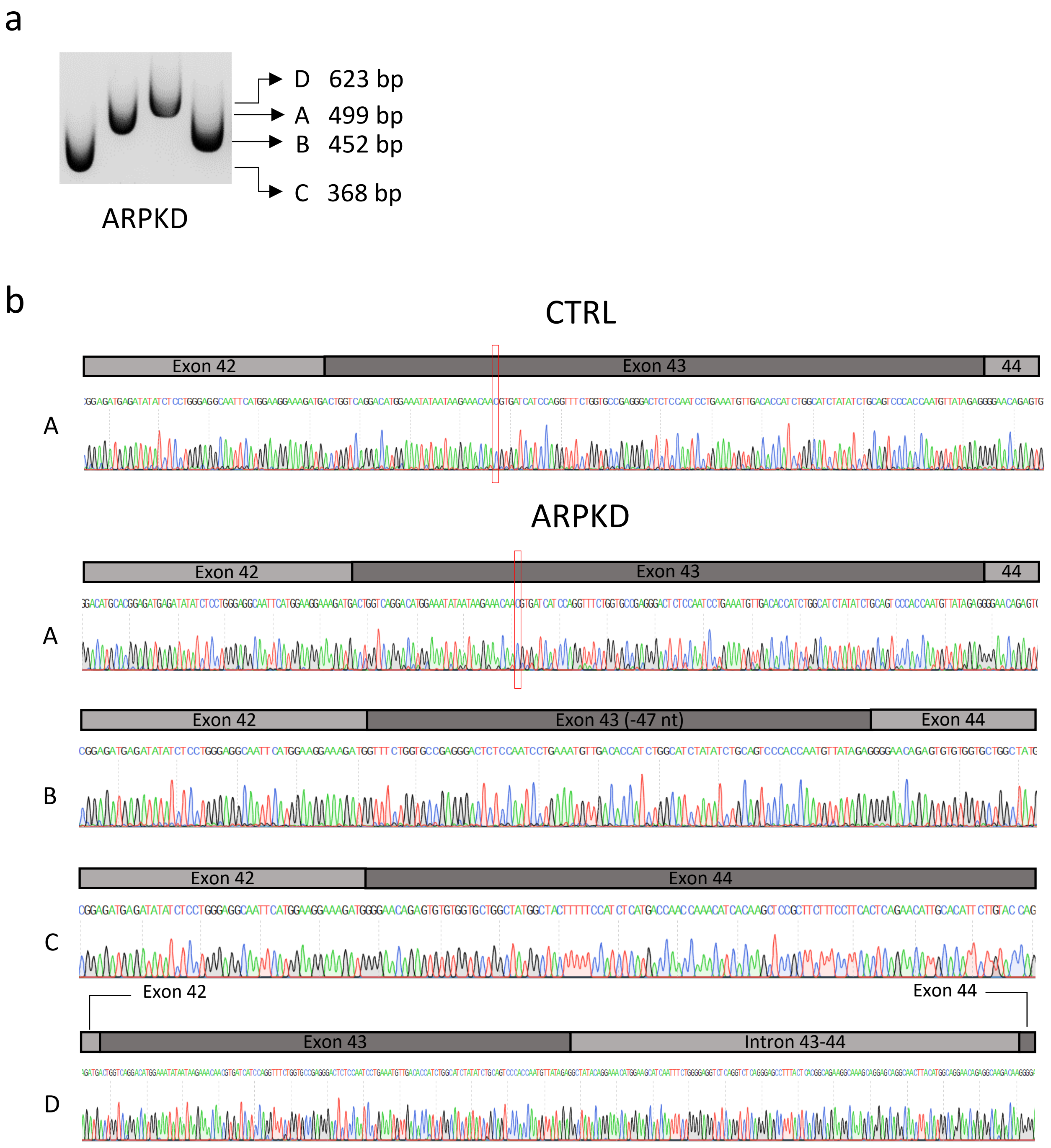

Additional Figure 3. Colony PCR after T-cloning of RT-PCR products indicates the expression of 4 different *PKHD1* transcript isoforms around exon 43 in ARPKD URECs. (a) Agarose gel shows the presence of 4 bands (368 bp, 452 bp, 499 bp, 623 bp) corresponding to 4 different *PKHD1* transcripts around exon 43. **(b)** Chromatograms results of Sanger sequencing of colony PCR products (CTRL transcript A and ARPKD transcripts A, B, C and D).

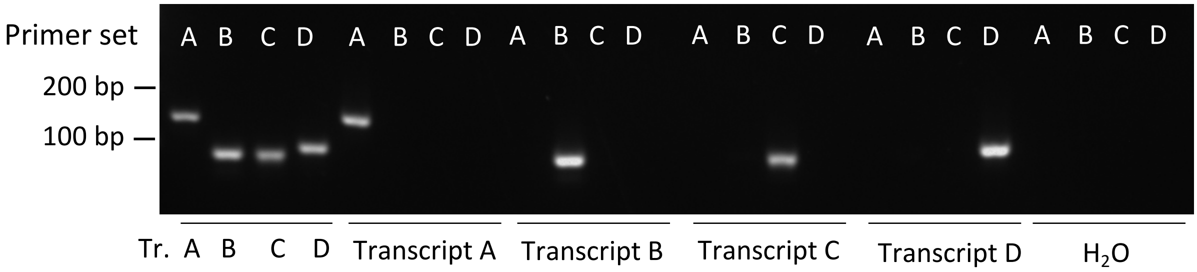

Additional Figure 4. PCR using transcript-specific primer sets shows the specificity of each primer pair to amplify only the corresponding colony PCR product. H_2O is used as negative control for the amplification reaction.

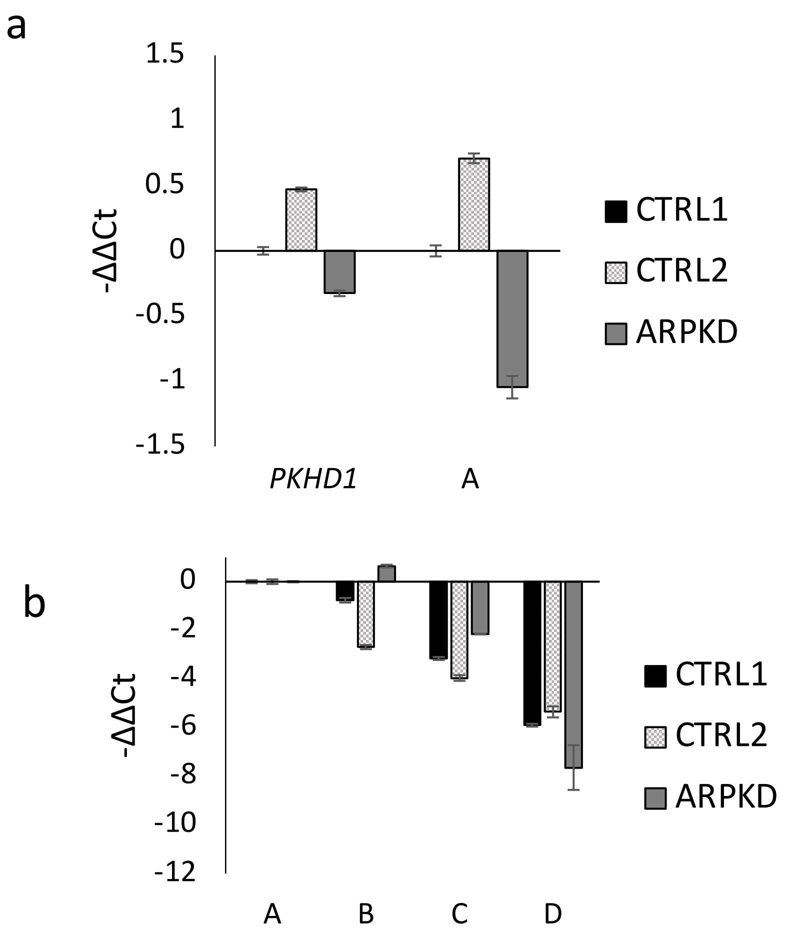

Additional Figure 5. Arithmetic mean of ΔΔCt values of 3 technical replicates for each sample from *PKHD1* expression analysis shown in Figures 2d and 2e. (a) Expression level of *PKHD1*, as measured by using a set of primers that do not differentiate between different splicing products around exon 43 and expression levels of transcript A in URECs from two different wild type controls and from ARPKD patient. Error bars represent the standard error between technical triplicates. Values are normalised to one of the two wild type controls. **(b)** Expression analysis of the different transcript isoforms (transcript B, C and D) around exon 43 of *PKHD1*, normalized to the expression levels of transcripts A, in URECs from two different wild type controls and from ARPKD patient. Error bars represent the standard error between technical triplicates.

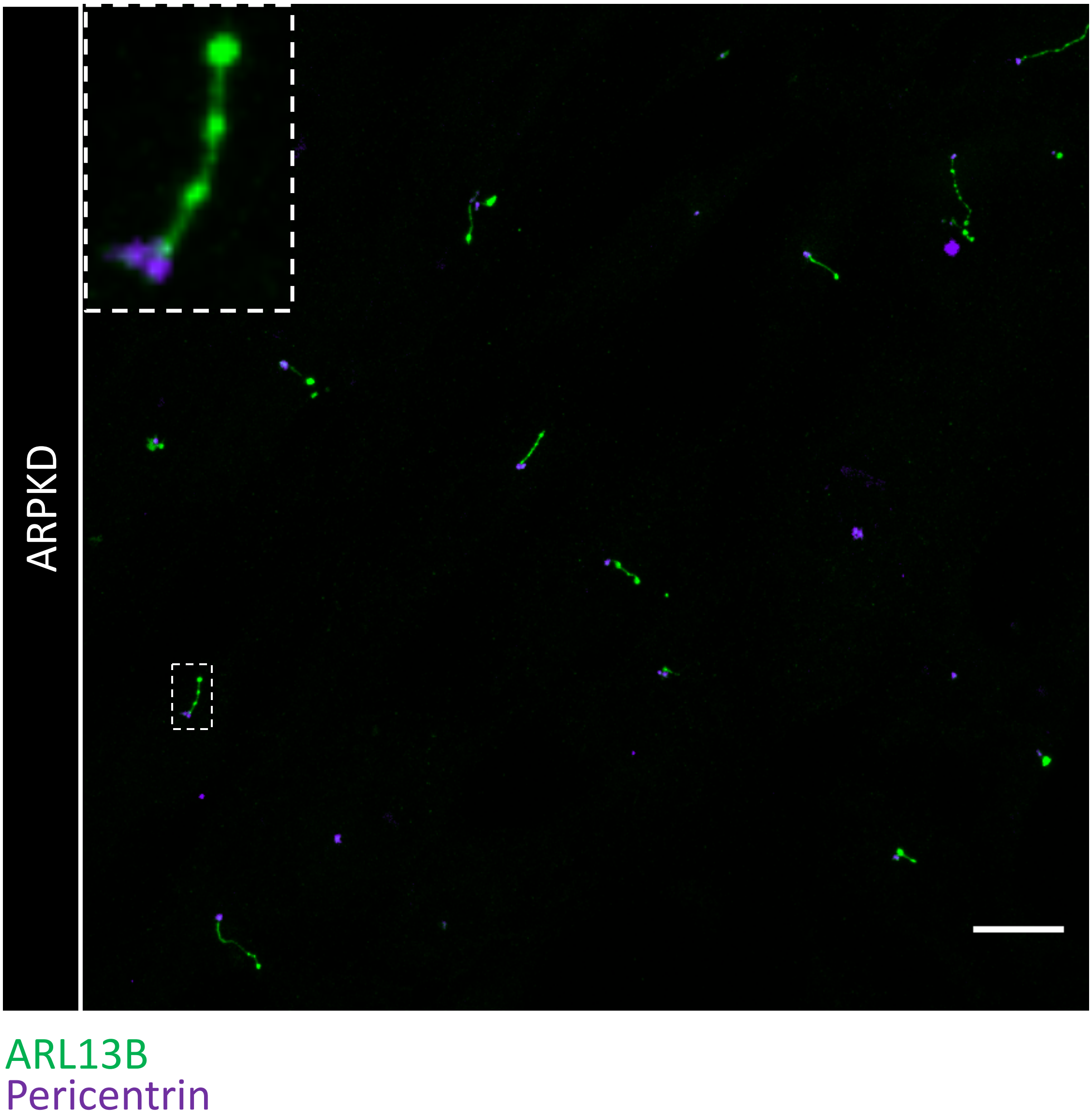

Additional Figure 6. Representative micrograph of ARPKD URECs. ARPKD cilia are characterised by blebs along the axoneme and at the ciliary tip, as demonstrated by co-staining of the ciliary membrane marker ARL13B, green and the basal body marker pericentrin, violet. Scale bar 10 µm. Inset, zoom-in of a representative cilium.

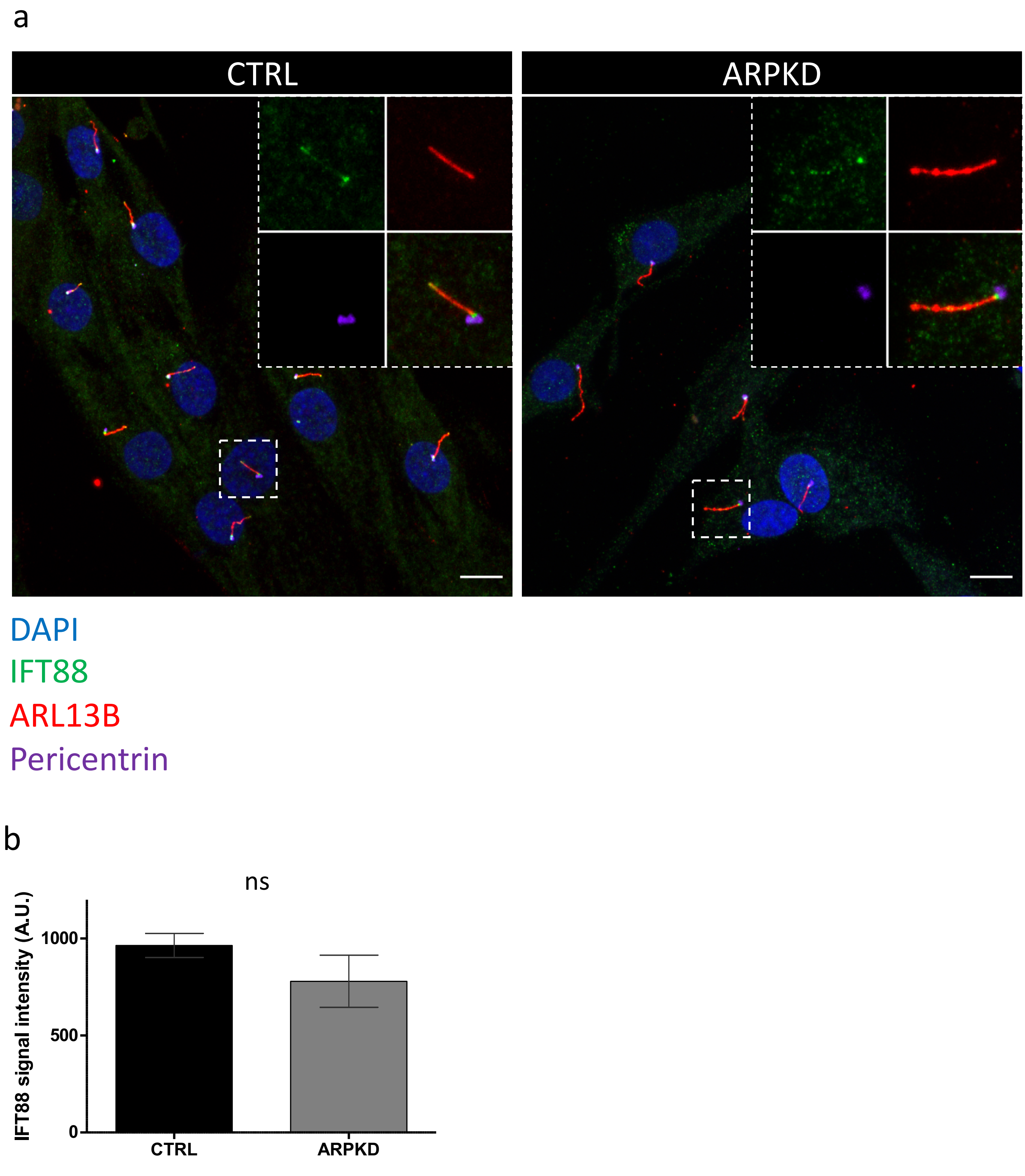

Additional Figure 7. IFT88 staining of CTRL and ARPKD URECs. (a) Representative immunofluorescence images of wild type control URECs and ARPKD URECs, serum starved for 48 h. No obvious defects in ciliary IFT88 localization is observed in ARPKD cells when compared to control. Blue – DAPI, Green – IFT88, Red – ARL13B, Violet, Pericentrin. Scale bar 10μm. Insets, zoom-in of representative cilia. **(b)** Quantification of IFT88 staining intensity in control and ARPKD URECs. Ns, non significant, n = 190. A.U., arbitrary units.

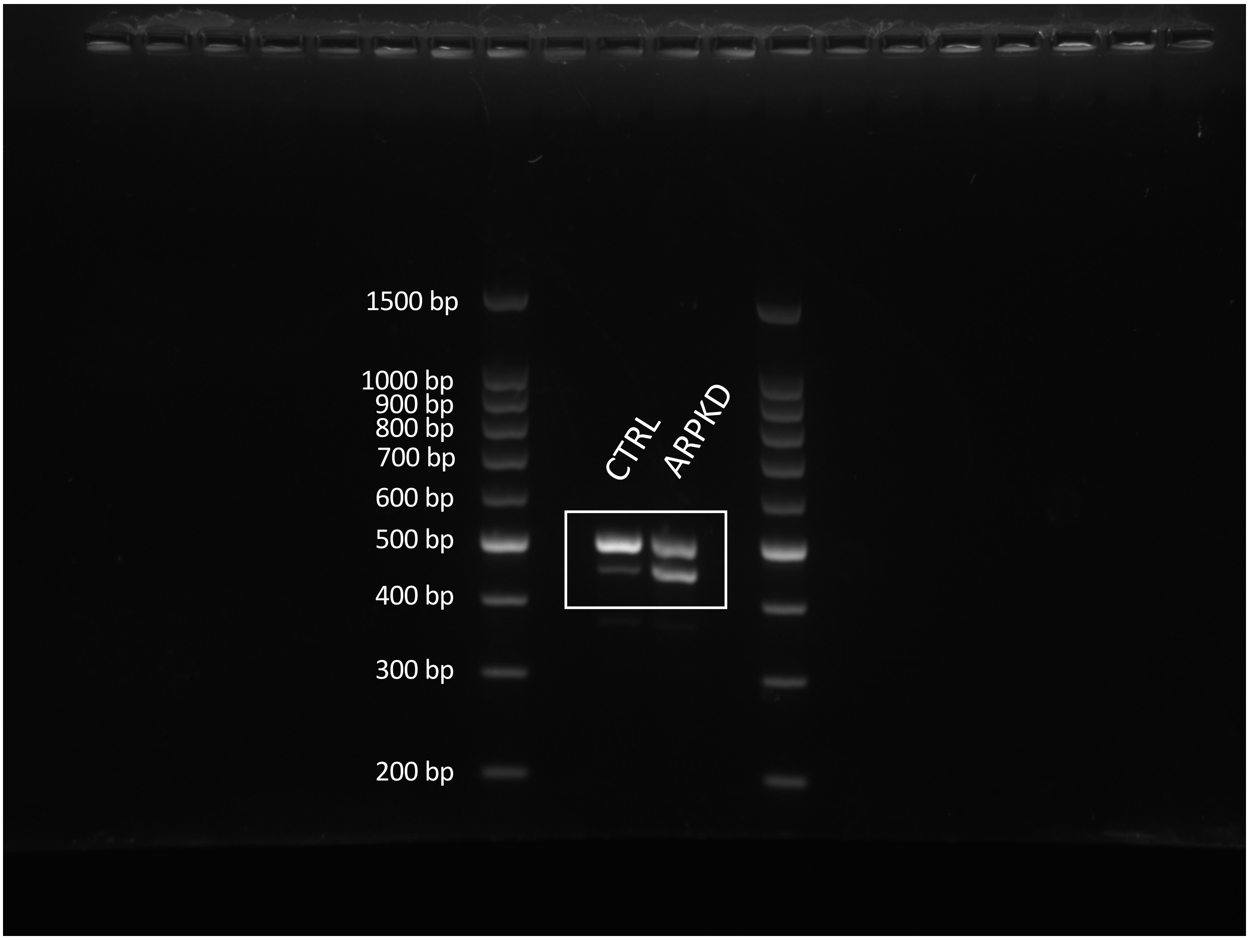

Additional Figure 8. Full gel image of RT-PCR on cDNA isolated from wild type control URECs and ARPKD URECs lysates (Figure 2b), demonstrating alternate splicing of exon 43 in both cell lines.

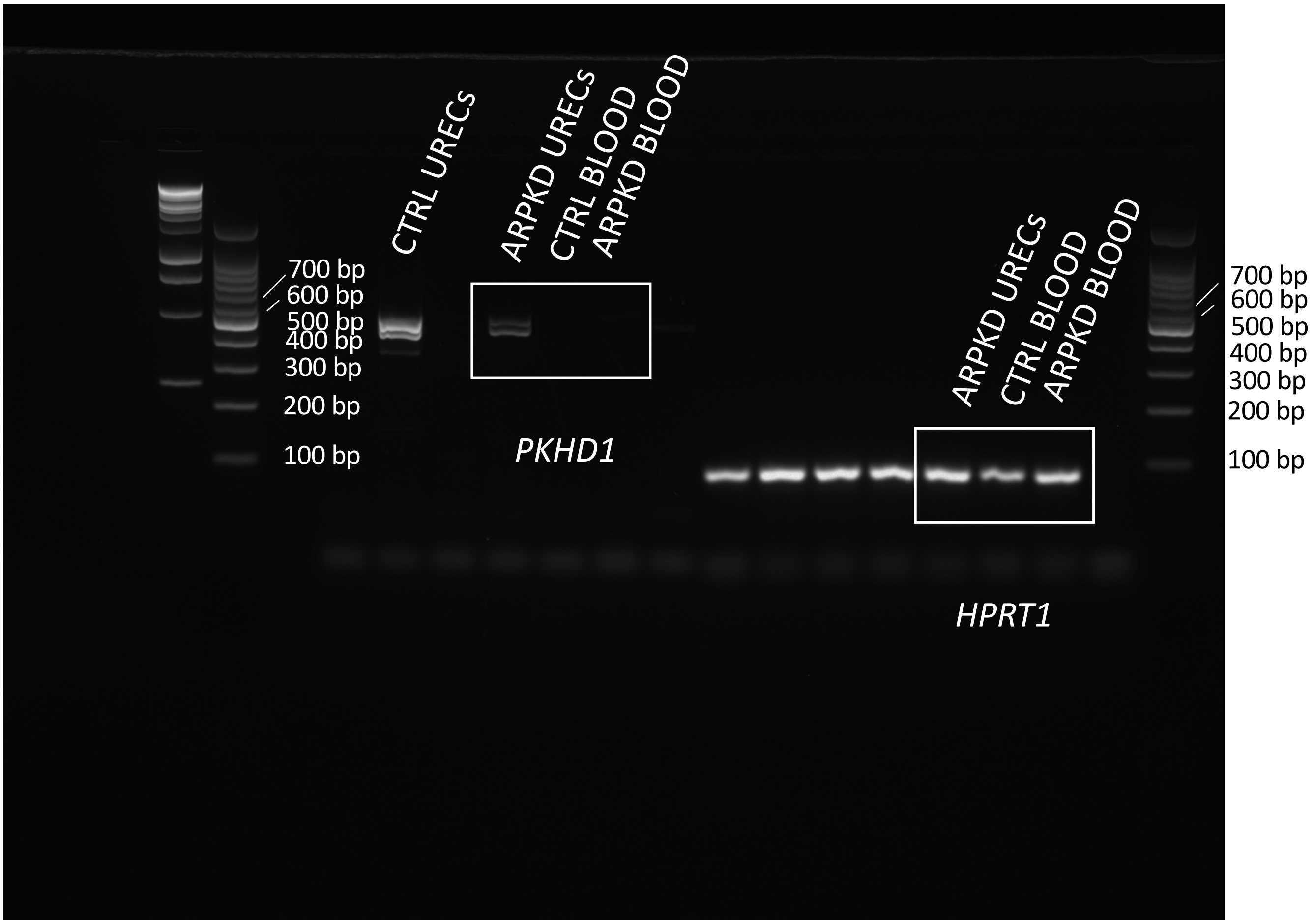

Additional Figure 9. Full gel image of RT-PCR on URECs and blood cDNA (Additional Figure 1). *PKHD1* is expressed at low levels in lymphocytes (blood) and fails to be amplified by RT-PCR on ARPKD blood cDNA, but it is expressed at a detectable level in URECs.

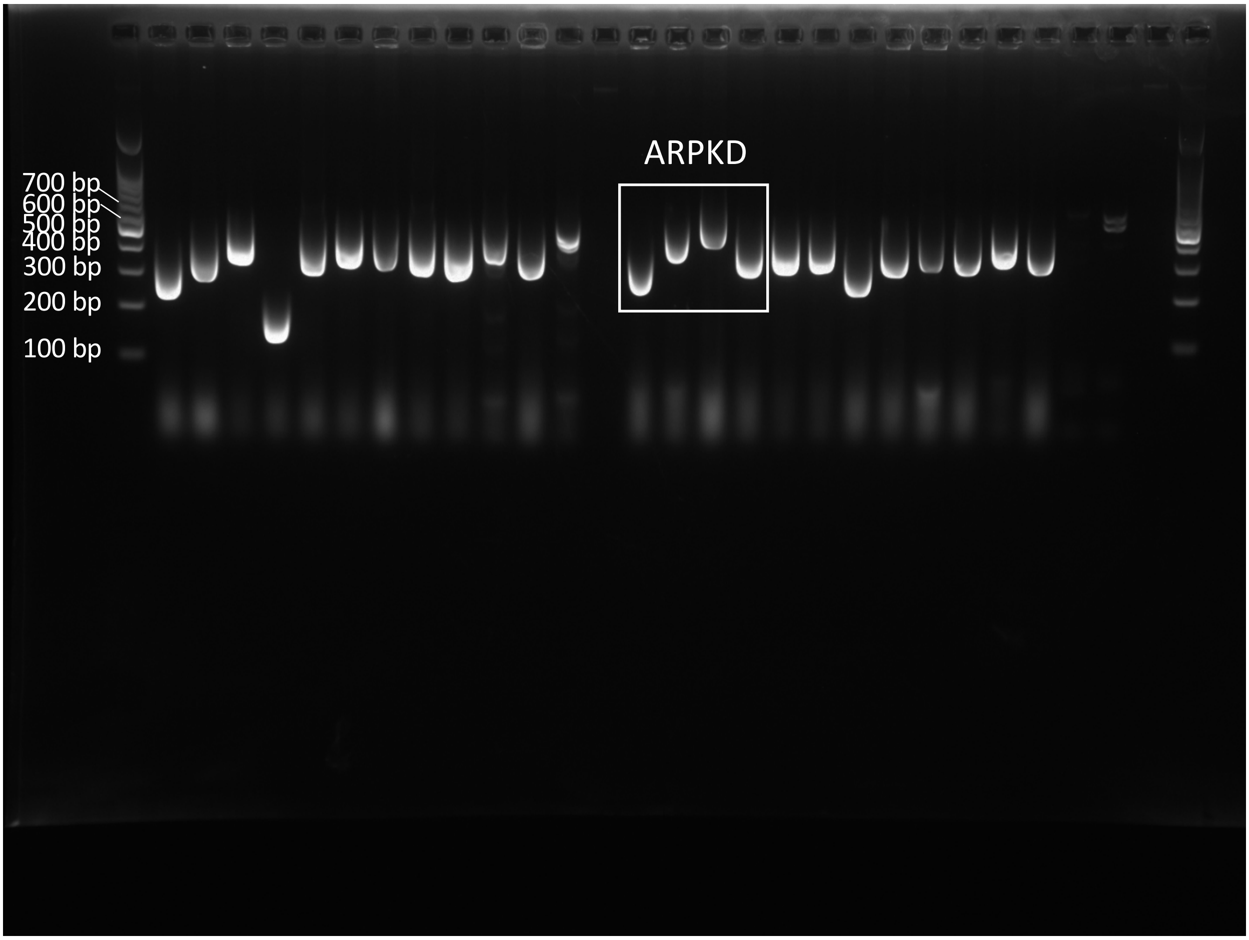

Additional Figure 10. Full gel image of colony PCR after T-cloning of RT-PCR products (Additional Figure 3), showing the expression of 4 different *PKHD1* transcript isoforms around exon 43 in ARPKD.

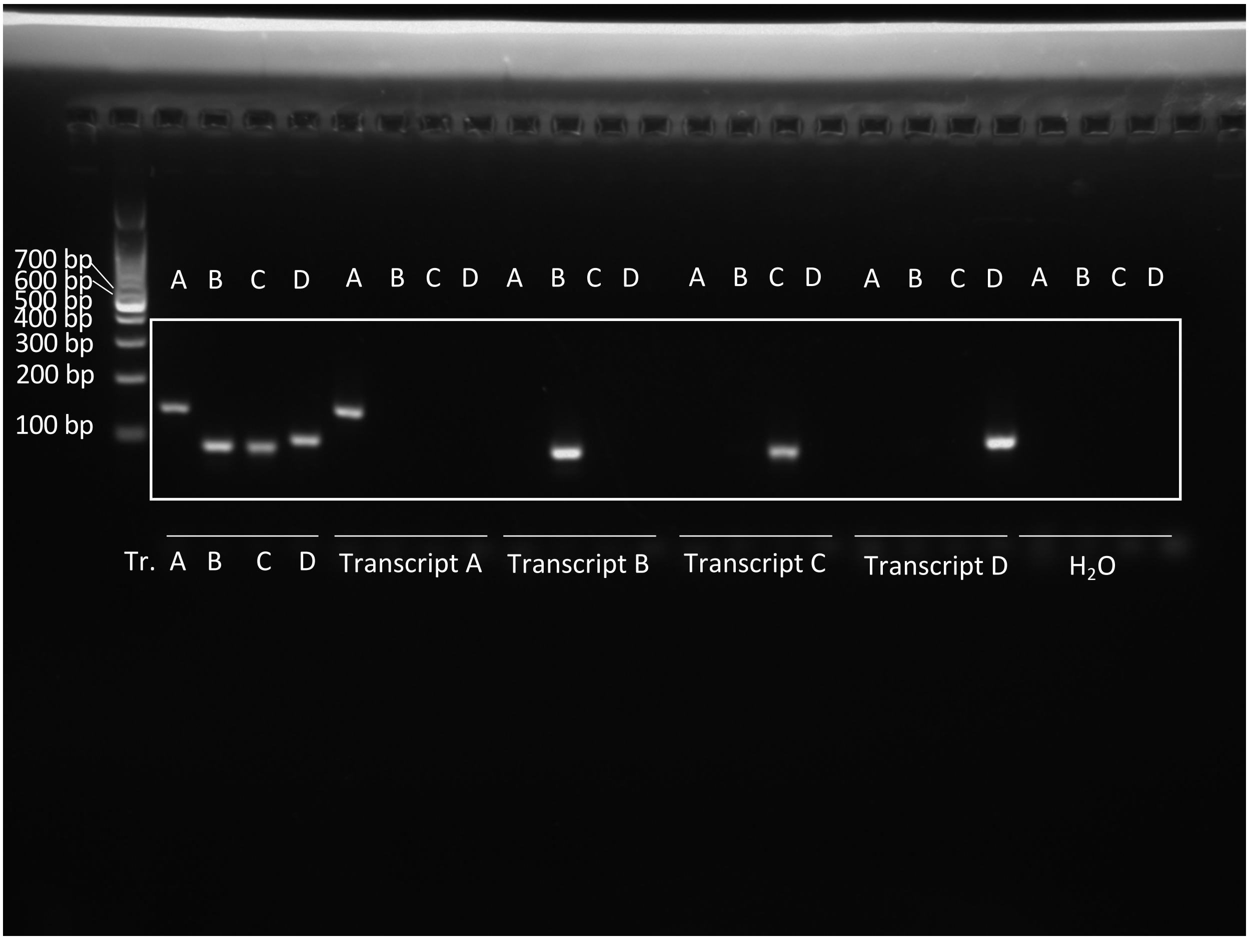

Additional Figure 11. Full gel image of PCR with transcript-specific primer sets (Additional Fig[illegible]3). Each primer pair specifically amplifies only the corresponding colony PCR product. H_2O is used as negative control for the amplification reaction.
